# One-Lung Ventilation Duration Is a Risk Factor for Pneumonia in Minimally Invasive and Robotic Esophagectomy

**DOI:** 10.3390/jcm15103832

**Published:** 2026-05-15

**Authors:** Vladimir J. Lozanovski, Julian Kobler, Edin Hadzijusufovic, Franziska Renger, Christoph Wandhoefer, Eva-Verena Griemert, Hauke Lang, Peter P. Grimminger

**Affiliations:** 1 Department of General, Visceral and Transplantation Surgery, University Medical Center of the Johannes Gutenberg University Mainz, Langenbeckstr. 1, 55131 Mainz, Germany; vladimir.lozanovski@unimedizin-mainz.de (V.J.L.);; 2 Department of Anesthesia, University Medical Center of the Johannes Gutenberg University Mainz, 55131 Mainz, Germany

**Keywords:** pneumonia, esophageal surgery, minimally invasive surgery, robotics, Da Vinci robot-assisted esophagectomy, Ivor Lewis esophagectomy, RAMIE, MIE, lung ventilation

## Abstract

**Introduction:** Postoperative pulmonary complications, particularly pneumonia, remain frequent after esophagectomy and contribute significantly to morbidity. One-lung ventilation (OLV) is a potential modifiable risk factor, but its impact in minimally invasive (MIE) and robot-assisted Ivor Lewis esophagectomy (RAMIE) within European populations is not well defined. **Methods:** 619 patients undergoing MIE or RAMIE were analyzed. OLV duration was extracted from operative records. Postoperative pneumonia incidence, overall survival, and perioperative outcomes were assessed. ASA classification and other risk factors were considered. **Results:** The overall incidence of postoperative pneumonia was 18.6%, with no significant difference between MIE (20.4%) and RAMIE (18.2%). Prolonged OLV duration increased pneumonia risk by 4% per 10 min. Female sex and higher ASA classification were also significant risk factors. Likely reflecting early diagnosis and advanced perioperative management, pneumonia did not affect overall survival, which remained comparable between MIE and RAMIE. **Conclusions:** Prolonged OLV during MIE and RAMIE increases the risk of postoperative pneumonia without significantly affecting overall survival, reflecting effective complication management. OLV duration may serve as a practical intraoperative indicator to guide risk stratification and optimize postoperative care in minimally invasive and robot-assisted Ivor Lewis esophagectomy.

## 1. Introduction

Esophageal cancer is among the most prevalent malignant tumors worldwide, with an estimated 500,000 new cases annually. It ranks seventh in cancer-related mortality. Adenocarcinomas account for most cases, followed by squamous cell carcinomas. The overall 5-year survival remains approximately 25% [1,2,3,4].

Surgical resection is the cornerstone of curative treatment. Standard management includes esophagectomy with systematic lymphadenectomy to achieve R0 resection. The abdominothoracic approach may be performed via open, hybrid, minimally invasive, or robot-assisted techniques, with minimally invasive or hybrid procedures recommended whenever feasible [5,6].

Esophagectomy is a technically demanding two-cavity operation associated with morbidity rates of 30–50% even in high-volume centers [7]. Postoperative complications substantially contribute to mortality, prolonged hospitalization, and increased healthcare costs. Pulmonary complications and anastomotic leakage are the most common postoperative complications and the strongest predictors of postoperative death [8].

Pulmonary complications occur in up to 30% of patients, depending on the cohort and definition used [9]. Several preoperative factors, such as diabetes, dyspnea, chronic obstructive pulmonary disease (COPD), and alcohol consumption, have been linked to an elevated risk [10]. In minimally invasive procedures, intraoperative parameters such as one-lung ventilation (OLV) have also been identified as relevant contributors to postoperative pneumonia [11]. OLV is preferred in minimally invasive and robot-assisted approaches although it has been shown to induce lung parenchymal injury through multiple pathophysiological mechanisms [12]. In McKeown esophagectomy, longer OLV duration has been associated with a higher incidence of postoperative pneumonia [12]. Yet, data assessing OLV duration as an independent risk factor in European cohorts predominantly undergoing robot-assisted or minimally invasive Ivor Lewis esophagectomy remain limited.

The objective of this study was to assess whether the duration of OLV affects the occurrence of postoperative pneumonia after minimally invasive (MIE) and robot-assisted minimally invasive esophagectomy (RAMIE).

## 2. Patients and Methods

This retrospective observational study included all 631 consecutive patients who underwent surgery for esophageal cancer at the Department of General, Visceral, and Transplantation Surgery, University Medical Center Mainz, between February 2015 and July 2023. The date of surgery was considered the index date. All procedures were performed by a consistent surgical team within a single institution, ensuring standardization of the procedure. Data were prospectively collected and systematically documented in an institutional database. Comprehensive follow-up was performed for all patients. All patients provided informed consent permitting anonymous data and follow-up collection, with potential use for scientific analysis. In accordance with federal state regulations (state hospital laws §36 and §37) and the independent ethics committee, no ethical approval was required for this study.

Inclusion criteria—beyond the diagnosis of esophageal cancer—were availability of operative duration, subdivided into thoracic and abdominal components; documentation of the surgical technique used; and recording of postoperative complications. Based on these criteria, 12 patients were excluded due to missing thoracic operative time, which could not be reliably extracted from the operative report, anesthesia record, or other medical documentation. Thus, a total of 619 patients were included in the final analysis.

### 2.1. Clinical, Pathological, and Survival Outcomes

The baseline dataset included demography, age, sex, and body mass index (BMI).

Preoperative parameters included American Society of Anesthesiologists physical status (ASA) classification, cardiac, pulmonary, hematologic, vascular, and neurological comorbidities, as well as specific conditions such as abdominal aortic aneurysm, dementia, COPD, peptic ulcer, liver cirrhosis, chronic kidney disease, human immunodeficiency virus (HIV) infection, diabetes, hemiplegia, prior abdominal or thoracic surgery, depression, asthma, myocardial infarction, and smoking.

Preoperative therapy data included information on neoadjuvant treatment, the type of neoadjuvant therapy, endoscopic resection, immunotherapy and the specific agent used, and the neoadjuvant regimen.

Surgical data included total operative time, thoracic and abdominal operative time, tumor location, type of surgery, surgical technique (MIE, RAMIE using the Da Vinci robotic system (Intuitive Surgical Inc., Sunnyvale, CA, USA), or hybrid), anastomotic site, type and technique of the anastomosis, anastomotic reinforcement, type of reconstruction, stapler size, creation of an omental flap and pleural tent, and intraoperative blood loss.

Pathology data comprised histology, cTNM, clinical Union for International Cancer Control (UICC) stage, pTNM, pathological UICC stage, number of harvested and positive lymph nodes, R-status, and R1 localization. Documentation included whether conversion occurred during the thoracic or abdominal phase, the reason for conversion, and any intraoperative complications.

Perioperative complications were classified according to the Esophagectomy Complications Consensus Group (ECCG) [13]. Complications included bleeding, anastomotic leakage, pneumonia, other pulmonary complications, pneumothorax, pleural effusion, pylorospasm, pulmonary embolism, need for reintubation or tracheostomy, recurrent laryngeal nerve palsy, chylothorax, atrial fibrillation, other cardiac complications, wound infection, sepsis, empyema, urinary tract infection, delayed gastric emptying, anastomotic stricture, Clavien–Dindo classification, intensive care unit (ICU) admission, and hospital readmission within 30 days.

Survival data comprised occurrence and site of metastases, disease-free survival, death, time of death, last follow-up, lost-to-follow-up status, 30-day in-hospital mortality (IHM), and overall survival from surgery and from diagnosis. Specifically, disease-free survival was defined as the length of time after primary treatment during which a patient survived without any signs of cancer recurrence (local or regional recurrence or distant metastasis). Overall survival was defined as the length of time from the date of surgery until death.

### 2.2. Definition of OLV

The primary parameter investigated in this study was the OLV duration during the thoracic phase of esophagectomy. Thoracic operative time was used as a surrogate marker because OLV occurs exclusively during this phase. Documentation was based on the interval from thoracic skin incision, which is immediately followed by trocar placement and creation of a pneumothorax, to skin closure. This method does not overestimate OLV duration, as the lung is typically re-ventilated shortly before skin closure. Moreover, prolonged thoracic operative time reflects complex or technically challenging intrathoracic steps rather than standardized wound closure, and a longer thoracic phase is inherently associated with extended OLV. This establishes a direct pathophysiological link between thoracic operative time and OLV duration, justifying its use as a surrogate marker in this study while accounting for the clinical setting. Also, this provides a simple, reproducible parameter that can be reliably obtained from multiple sources, including electronic and written intraoperative records, and anesthesia documentation.

### 2.3. Definition of Pneumonia

Pneumonia within the first two weeks after esophagectomy was defined according to the ECCG and local guidelines and diagnosed based on clinical findings and imaging [13,14]. Infiltrates were diagnosed radiologically, along with at least one clinical criterion: leukocytosis or leukopenia, fever, or purulent secretions [13,14].

### 2.4. Statistical Analysis

Statistical analyses were performed using SPSS (IBM Corp., 2015, IBM SPSS Statistics for Windows, Version 29.0, Armonk, NY, USA) and R version 4.2 (R, 2024, (http://www.R-project.org accessed on 4 December 2025)). Categorical variables were reported as absolute and relative frequencies. Continuous variables were summarized using the mean and standard deviation, with minimum, maximum, sample size, and percentiles reported when appropriate. Group differences were primarily assessed using nonparametric tests, which do not assume normal distribution or homogeneity of variance. For categorical data, the Chi-square test or Fisher’s exact test (Fisher-Yates test for small sample sizes) was used. Differences in continuous variables between two groups were evaluated using the Mann–Whitney U test. For comparisons involving more than two groups or general analysis of continuous variables, rank-based analysis of variance was applied. Associations between risk factors and relevant endpoints were analyzed using logistic regression, which was also used to develop the predictive model. Results are presented as odds ratios (OR) with corresponding 95% confidence intervals (CI). Multivariate logistic regression, adjusting for relevant covariates, was visualized using forest plots. A receiver operating characteristic (ROC) analysis with Youden’s index was performed to identify a cut-off value for thoracic operative time (OLV duration) for prediction of postoperative pneumonia. Survival times were estimated using the Kaplan–Meier method, with median, mean, standard error, and interquartile range reported. Post hoc power analysis was performed, assuming a two-sided α of 0.05, to detect small survival differences and a potential risk of type II error. Differences between groups were tested using the log-rank test, with results expressed as *p*-values. Kaplan–Meier curves were used for graphical presentation. Multivariate survival prediction was performed using Cox proportional hazards regression, with hazard ratios (HR), 95% confidence intervals, and *p*-values reported. A *p*-value < 0.05 was considered statistically significant.

## 3. Results

Baseline demographic, perioperative, and postoperative characteristics of the 619 patients who underwent esophagectomy are summarized in Table 1. Most patients underwent Ivor Lewis procedures, with RAMIE performed in 53%, MIE in 37%, and open or hybrid approaches in 10%. The cohort had a mean age of 64 years and was predominantly male, and most patients were classified as ASA II–III. The majority presented with adenocarcinoma and received neoadjuvant therapy. Postoperative anastomotic leakage occurred in 11.8% of patients.

No significant differences were observed between groups for most baseline variables. Prior abdominal surgery was more frequent in some groups, reflecting its influence on the choice of surgical approach. RAMIE showed longer thoracic and overall operative times compared with MIE, while open and hybrid approaches had longer abdominal phases. Despite this, both total and ICU stay were significantly shorter after RAMIE (*p* < 0.05), with comparable complication rates across all groups (Table 1).

### 3.1. Time Distribution of Different Surgical Approaches

Table 2 illustrates the distribution of operative techniques across hourly intervals of thoracic operative time. A significant association was found between operative approach and duration (*p* < 0.001). Most open/hybrid procedures (59.3%) lasted 2–3 h, whereas most RAMIE cases (48.8%) ranged from 3–4 h. The MIE group showed a balanced distribution between 2–3 h (37.8%) and 3–4 h (41.5%). For procedures exceeding 4 h, MIE (17.4%) and RAMIE (17.3%) showed similar proportions, while open/hybrid procedures accounted for fewer cases (8.5%). Shorter procedures (<2 h) were mainly observed in MIE (10%) (Table 2). These findings indicate distinct duration patterns for each surgical approach and explain the significant differences observed in Table 1.

### 3.2. Incidence of Pneumonia in Relation to OLV

**Figure 1:**
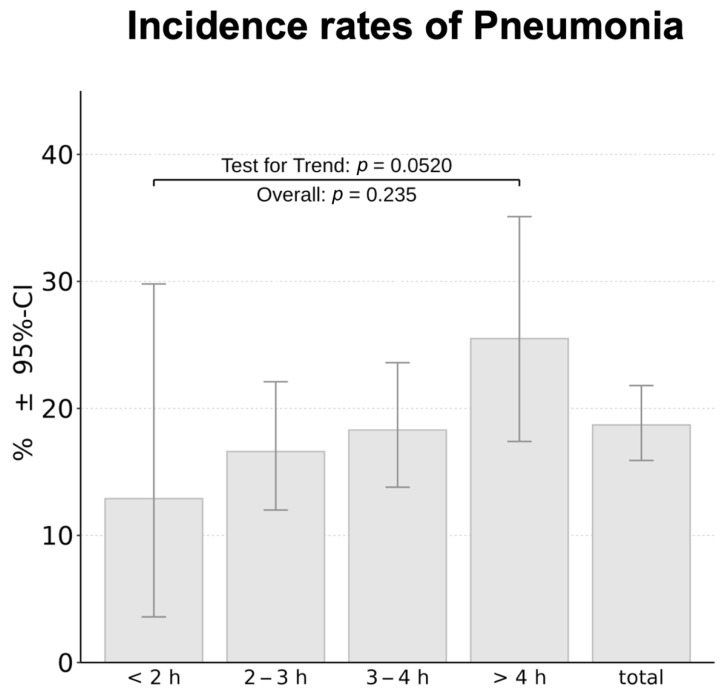
Incidence of pneumonia by one-lung ventilation duration [h] on the x-axis with 95% CI.

The established time categorization was used to further investigate whether OLV duration influences the incidence of postoperative pneumonia. The incidence of pneumonia in different time categories is shown in Figure 1. The analysis was based on the entire cohort of 619 patients, without stratification by surgical approach (Table 3). Pneumonia rates increased progressively with longer operative time categories. In procedures lasting less than 2 h, pneumonia occurred in 12.9% of cases, while in those exceeding 4 h, the incidence rose to 25.5%. The overall pneumonia rate in the total cohort was 18.6%. Across all time categories, a positive trend test using logistic regression indicated a potential association between longer OLV duration and the occurrence of postoperative pneumonia. In addition, a Cochran-Armitage test for trend was performed to assess the observed trend. The analysis demonstrated a borderline statistically significant trend toward increasing postoperative pneumonia with longer OLV duration (*p* = 0.0512), supporting the observed monotonic increase in pneumonia incidence across time intervals.

### 3.3. Longer OLV Duration Is Associated with an Increased Risk of Pneumonia

The observed association between OLV duration and pneumonia incidence raises the question of whether thoracic operative time may serve as a predictive parameter for postoperative pneumonia. This analysis focused on the MIE and RAMIE groups, as both procedures involve a capnopneumothorax with an iatrogenic increased intrathoracic pressure and are therefore comparable. Logistic regression analysis of 560 (230 MIE and 330 RAMIE) cases demonstrated a significant predictive effect (*p* = 0.026). The corresponding odds ratio indicated that the risk of postoperative pneumonia increases by a factor of 1.004 (95% CI: 1.0005–1.0083) per minute of thoracic operative time, corresponding to a 0.4% increase in risk per minute and approximately a 4% increase for every 10 min of prolonged OLV. Moreover, OLV duration demonstrated a borderline statistically significant association with postoperative pneumonia (OR 1.276, 95% CI: 0.998–1.662), suggesting a trend toward increased risk with longer OLV duration. A receiver operating characteristic (ROC) analysis with Youden’s index was performed, identifying a cut-off value of 212 min for OLV duration for prediction of postoperative pneumonia. This threshold demonstrated moderate sensitivity and specificity, supporting its potential use as a pragmatic intraoperative reference point for risk stratification rather than a strict clinical cutoff.

**Figure 2:**
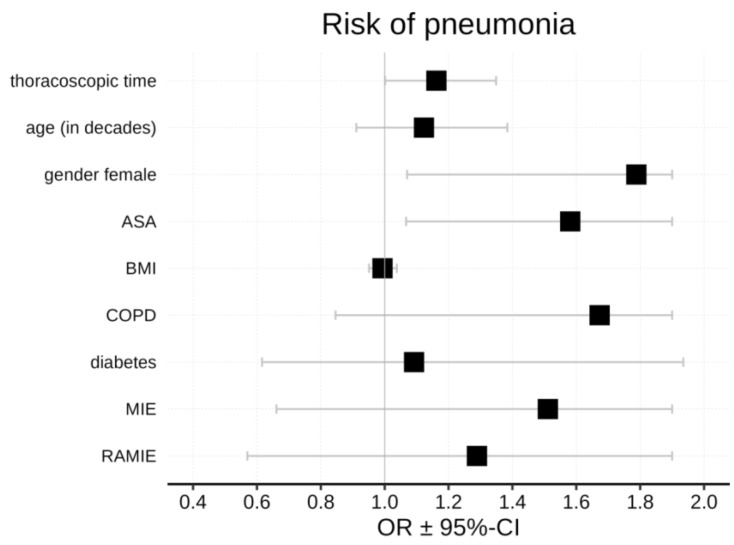
Forest plot of pneumonia incidence with 95% CI and predictability based on one-lung ventilation duration (shown in 30-min increments) and other factors. Black squares represent the OR estimates.

To identify additional risk factors, a forest plot was used, adjusting the primary study parameter (OLV) for sex, ASA classification, BMI, COPD, diabetes, MIE, and RAMIE. OLV again indicated an increased risk of pneumonia, as the point estimate suggested a positive effect (Figure 2). Taken together with the logistic regression results, these findings indicate that both OLV duration categorized in half-hour intervals and the continuous increase in operative time are associated with a higher risk of postoperative pneumonia. Key potential confounders, including smoking status, pulmonary comorbidities, and neoadjuvant therapy, were included in additional adjusted logistic regression models. The association between longer OLV duration and postoperative pneumonia remained consistent after adjustment (*p* = 0.0167; OR 1.4061, 95% CI 1.0637–1.8587). Smoking status, pulmonary comorbidities, and neoadjuvant therapy were not significantly associated with postoperative pneumonia in this model (all *p* = n.s.) suggesting that the observed effect of OLV duration was not substantially confounded by these variables.

### 3.4. Postoperative Pneumonia Incidence in MIE and RAMIE

The RAMIE and MIE groups were analyzed for potential differences in postoperative pneumonia rates, with no significant difference observed between the groups (*p* = 0.505).

**Figure 3:**
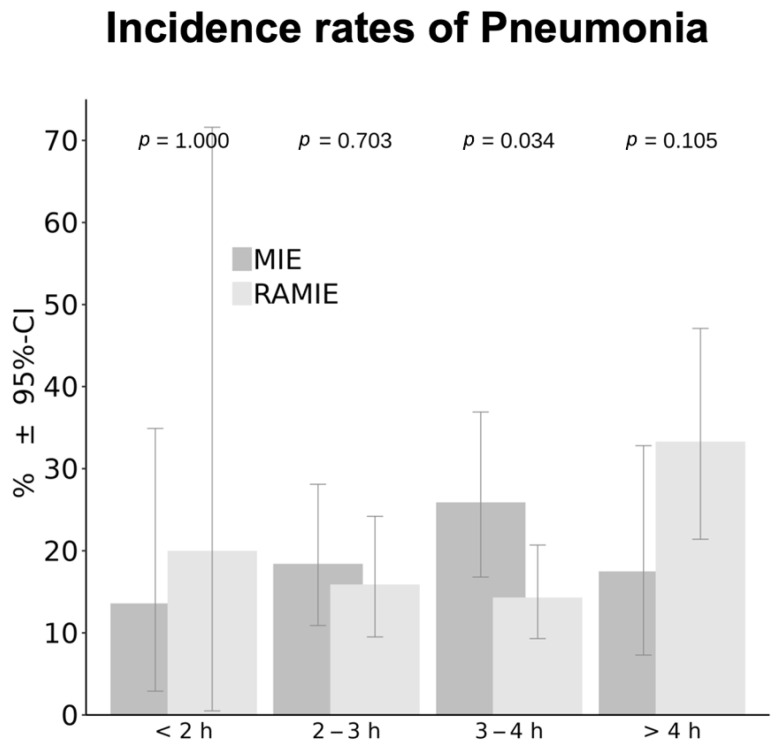
Incidence of pneumonia by surgical approach and one-lung ventilation duration [h] with 95% CI.

When examining pneumonia incidence according to the established time intervals, opposite trends were noted between the two groups. In the MIE group, pneumonia rates increased up to the 3–4-h interval and then declined in procedures longer than 4 h. In contrast, the RAMIE group showed decreasing incidence up to 3–4 h, followed by an increase in procedures exceeding 4 h. This pattern forms two opposing parabolas: an upward-opening curve for RAMIE and a downward-opening curve for MIE. Fisher’s exact test revealed a significant difference between MIE and RAMIE in the 3–4-h interval (*p* = 0.0034). To explore the observed group difference in the 3–4-h interval shown in Figure 3, the ASA classification was examined in more detail. As noted above and shown in Figure 2, ASA is a known risk factor for postoperative pneumonia. In this subgroup analysis, ASA I and II were combined as a “low risk” category and ASA III and IV as a “high-risk” category, due to the small numbers of ASA I (*n* = 1) and ASA IV (*n* = 8) patients. Analysis showed that significantly more patients in the MIE group within the 3–4-h interval were classified as high risk (ASA III–IV vs. ASA I–II; *p* = 0.026), which may partially explain the observed difference between groups. Higher ASA classification (ASA III–IV) was confirmed as an independent predictor of postoperative pneumonia in multivariable analysis (*p* = 0.0034; OR 1.9115, 95% CI 1.2390–2.9491), together with OLV duration in this interval (*p* = 0.0419). Although ASA distribution differed between MIE and RAMIE in this subgroup, interaction analysis did not demonstrate a statistically significant interaction between ASA status and OLV duration (*p* = 0.0768), suggesting no relevant effect modification. To further explore potential differences between surgical approaches, interaction analyses were performed. No statistically significant interaction was found between ASA classification and surgical technique (MIE: *p* = 0.2488; RAMIE: *p* = 0.2342), nor between ASA status and OLV duration, suggesting that the observed differences in pneumonia trends are not driven by effect modification of ASA.

With regard to other potential contributing factors, year of operation was not significantly associated with pneumonia risk (*p* = 0.1058). In contrast, higher intraoperative blood loss was significantly associated with an increased risk of postoperative pneumonia (*p* = 0.0032).

Regarding anastomotic technique, the cohort was largely homogeneous, with end-to-side anastomosis being the predominant approach; therefore, a meaningful comparative analysis of alternative techniques was not feasible.

### 3.5. Survival Analysis

**Figure 4:**
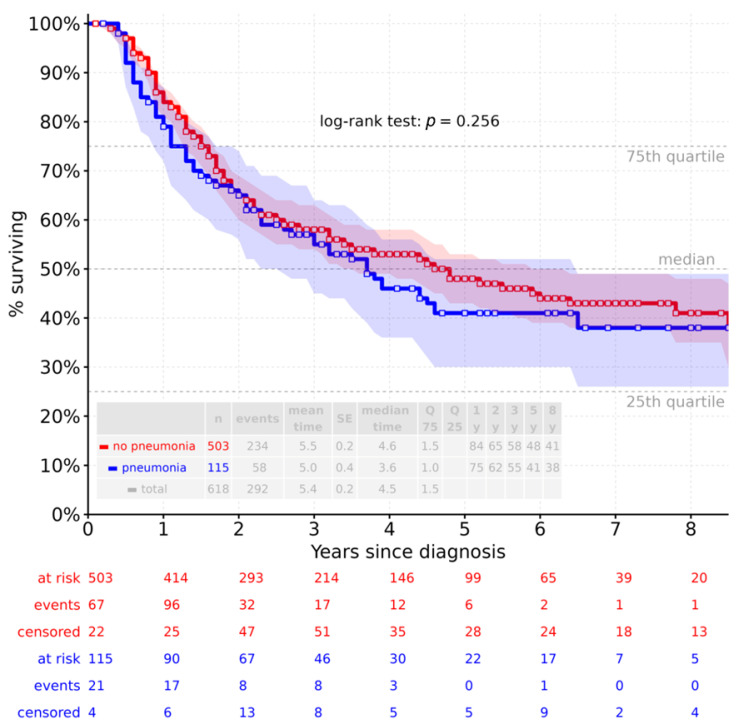
Overall survival (*n* = 619) according to pneumonia status.

The overall survival analysis showed a median survival of 4.5 years for the entire cohort (mean survival of 5.4 years). In patients who developed pneumonia, median survival was 3.6 years (mean survival 5.0 years), compared with 4.6 years (median) and 5.5 years (mean) in patients without pneumonia (Figure 4). The log-rank test revealed no significant difference in overall survival between patients with and without pneumonia (*p* = 0.256), indicating that postoperative pneumonia was not a statistically predictive factor for survival in this cohort.

**Figure 5:**
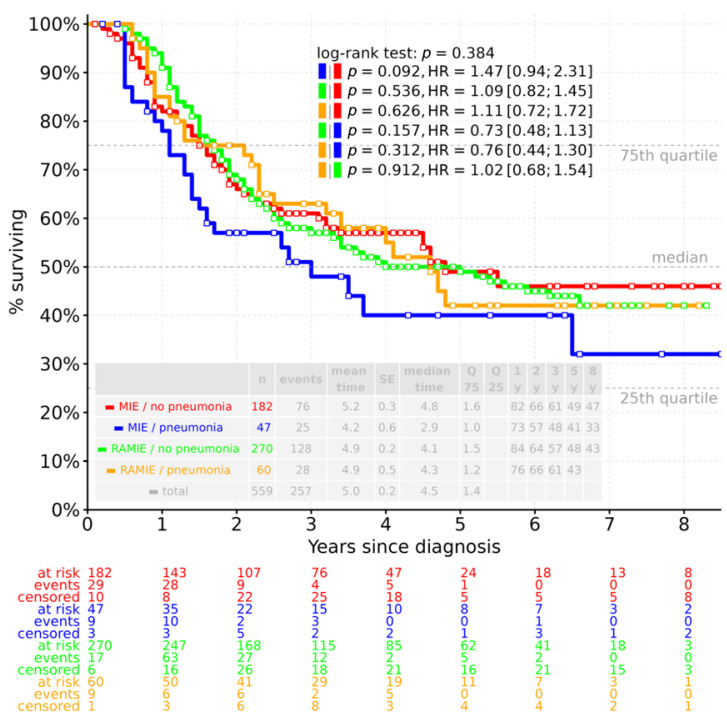
Overall survival (*n* = 619) according to pneumonia status and surgical approach.

Stratified by operative approach (MIE or RAMIE) and pneumonia incidence, median and mean survival were as follows: MIE without pneumonia, 4.8 and 5.2 years; MIE with pneumonia, 2.9 and 4.2 years; RAMIE without pneumonia, 4.1 and 4.9 years; RAMIE with pneumonia, 4.3 and 4.9 years. Log-rank comparisons between groups showed no significant differences (Figure 5). Overall, survival analysis revealed no statistically significant differences in overall survival between RAMIE and MIE or in relation to postoperative pneumonia.

Post hoc power analysis was performed, assuming a two-sided α of 0.05, an observed hazard ratio of 1.1849, and a group size ratio of 4.37 (503 vs. 115 patients). The resulting statistical power was 18.3%, indicating limited ability to detect small survival differences and a potential risk of type II error. In addition, competing risks were addressed using multivariable Cox regression. Recurrence and postoperative bleeding were identified as independent predictors of reduced overall survival (both *p* < 0.05), whereas other postoperative complications, including anastomotic leakage, pneumothorax, pleural effusion, and pylorospasm, were not significantly associated with survival.

## 4. Discussion

OLV duration appears to be a clinically relevant intraoperative parameter associated with postoperative pneumonia, supporting its potential role as a practical intraoperative marker for risk stratification. Prolonged OLV during MIE and RAMIE increases the risk of postoperative pneumonia, with a 4% rise in risk per 10 min. Moreover, the overall pneumonia rate of 18.6%, an anastomotic leakage rate of 11.8%, and a 30-day mortality rate of 1.5% in this study were all lower than the benchmark values of 25.7%, 15.9%, and 0.9–2.6%, respectively [15]. These findings indicate that European high-volume centers can achieve results comparable to, or even exceeding, established international standards.

OLV refers to the functional separation of both lungs, in which one lung is intentionally collapsed and not ventilated, while the other is ventilated and maintains blood oxygenation. This is achieved by using a double-lumen endotracheal tube or bronchus blocker that allow selective lung ventilation [16]. OLV facilitates surgical exposure during abdominothoracic esophagectomy by enabling complete collapse of the right lung, typically achieved with a left-sided double-lumen tube. This atelectatic state is preferred in both minimally invasive and robot-assisted approaches. Pathophysiologically, OLV results in an intrapulmonary right-to-left shunt, as deoxygenated blood from the non-ventilated lung returns to the left heart, potentially leading to a drop in oxygen saturation. Carbon dioxide elimination, however, can be compensated through the ventilated lung. Normally, shunt volume is markedly reduced by hypoxia-induced pulmonary vasoconstriction, which redirects perfusion toward the ventilated lung. As a result, only a small proportion of patients experience significant hypoxemia during OLV [17]. Experimental studies have demonstrated that OLV induces alveolar damage, inflammatory responses, and oxidative stress in both the ventilated lung and the atelectatic lung. Unilateral ventilation enhances the mechanisms of ventilation-induced lung injury, including alveolar overdistension and repeated alveolar collapse and reopening [18]. Further animal studies indicate that longer OLV duration correlates with progressive injury in both the collapsed and contralateral lungs [19]. Studies in lung resection have shown that OLV provokes an inflammatory response of the bronchial epithelium in both lungs, confirming the association between longer OLV duration and inflammatory activation. This pulmonary inflammation, characterized by cytokine release, may contribute to postoperative complications [20]. Another important but often overlooked factor is that the inflammatory response is modulated via the vagus nerve [21,22]. Frequent pulmonary complications and pneumonia after esophagectomy may be related to pulmonary vagotomy, as the vagus regulates key lung functions such as the cough reflex, mucus production, and bronchial tone and plays a critical role in inflammation [23]. This disruption can lead to sputum stasis and aspiration, with inflammation as the final common pathway, supported by studies linking postoperative systemic inflammatory response syndrome to pulmonary complications [24]. Preserving the pulmonary branches of the vagus nerve during esophagectomy may therefore be beneficial [25].

Clinical studies have demonstrated an association between OLV and postoperative pulmonary complications. Lai et al. identified OLV as a significant risk factor in patients undergoing McKeown esophagectomy [12]. Patients with OLV durations exceeding 150 min showed a significantly higher incidence of postoperative pneumonia and an increased risk of pulmonary complications, with (OR = 2.73, 95% CI: 1.57–4.74) [12]. Another study found that the use of a double-lumen endotracheal tube represents an independent risk factor for pulmonary complications [26]. However, these findings are derived from Asian cohorts and McKeown procedures, which limits their comparability and applicability to European populations. In a German multicenter study, the duration of OLV was identified as a significant risk factor for postoperative pulmonary complications. A cutoff value of 175 min was determined for patients undergoing thoracotomy for indications other than primary lung cancer, beyond which pulmonary complications, including pneumonia, occurred significantly more frequently [27]. Moreover, reported thoracic operation times, often used as a surrogate for OLV, vary across studies. Peters et al. found thoracic times of 195–241 min depending on the endpoint, while pneumothorax duration in MIE was reported at 224–245 min for patients with or without pulmonary events [11,28]. Comparisons across studies are limited due to differing endpoints, surgical techniques, and patient characteristics. Importantly, OLV duration has implications for patient selection and operative planning. Factors such as thoracic width influence thoracic operative time and, consequently, OLV duration [29]. Experience in robotic procedures affects operative efficiency, with learning curves showing a reduction in thoracic time over sequential cases [30,31,32].

Data on robot-assisted Ivor Lewis esophagectomy have primarily focused on predictors for anastomotic leakage rather than pneumonia, and cohort sizes were smaller, further highlighting the limited evidence base for OLV-specific risk in robot-assisted procedures [28]. Published postoperative pneumonia rates after robot-assisted Ivor Lewis esophagectomy range from 17–19% over various periods, and pulmonary complications after MIE have been reported at 22.2%, with no significant differences between techniques [33,34]. These rates are broadly consistent with published data, reinforcing that RAMIE and MIE share comparable pulmonary risk profiles, despite some heterogeneity in study cohorts and reported endpoints [15,34,35,36]. In our study, we observed an upward trend for RAMIE and a downward trend for MIE, with a significant difference between the two techniques in the 3–4-h interval. Analysis of ASA classification, a known risk factor for postoperative pneumonia, revealed a higher proportion of high-risk patients (ASA ≥ III) in the MIE group within this interval, which may partially explain the observed difference. Higher ASA classification was confirmed as an independent risk factor for postoperative pneumonia; however, interaction analyses showed no significant effect modification between ASA status and either surgical approach or OLV duration. These findings suggest that ASA reflects baseline patient risk rather than explaining the differential pneumonia patterns observed between MIE and RAMIE. Nevertheless, in patients with high ASA scores undergoing MIE, careful attention to prolonged OLV duration, ideally not exceeding 3–4 h, may be warranted.

Although OLV duration may partly reflect procedural complexity, it remained independently associated with postoperative pneumonia after adjustment for relevant comorbidities, neoadjuvant therapy, and baseline patient characteristics, supporting both a surrogate and a potential direct pathophysiological effect. Among other factors, higher intraoperative blood loss was identified as an additional risk factor, whereas the cohort was largely homogeneous regarding anastomotic technique, precluding meaningful comparative analysis. Overall, these findings suggest that the observed differences are primarily driven by patient-related rather than procedural factors. However, given the retrospective design, the findings should be interpreted as associative rather than causal and require prospective validation.

Regarding survival, most studies report no significant difference in overall survival between MIE and RAMIE [37]. Postoperative pulmonary complications, however, can moderately affect long-term outcomes, with some analyses showing a 5-year survival benefit for patients without pulmonary events [8,9,38]. Nevertheless, variations in study design, patient selection, and endpoints limit the comparability of these findings. In the present study, consistent with previous reports, pneumonia was not associated with reduced overall survival, and outcomes were comparable between MIE and RAMIE. Combined with the very low 30-day mortality, this likely reflects effective complication management and early diagnosis. The absence of survival differences should nevertheless be interpreted cautiously given the limited statistical power for this endpoint. In addition, multivariable survival analysis including recurrence and postoperative complications identified recurrence and postoperative bleeding as the main determinants of reduced survival, whereas other postoperative complications were not independently associated with outcome. Within this context, OLV has been identified as a potentially modifiable risk factor for postoperative pulmonary complications in MIE [11]. OLV duration is easily obtained from operative records, as shown in the present study, and may serve as a practical tool for perioperative risk assessment, while targeted strategies, including tailored antibiotic regimens, could further reduce pneumonia in high-risk patients [39]. Machine learning approaches to predict postoperative complications, as described by van de Beld et al., are challenging even when extensive preoperative and laboratory data are used; however, intraoperative OLV duration was not included in these models [40]. Incorporating this parameter into predictive models may further improve the prediction of postoperative pulmonary complications.

Limitations of the present study include its retrospective, observational, single-center design. In this study, OLV duration showed a statistically borderline predictive capability in logistic regression. Therefore, OLV duration should be interpreted as a clinically relevant and potentially predictive intraoperative parameter, rather than a definitive independent predictor. These findings support its use for risk stratification, while prospective studies are needed to confirm a causal relationship. Another limitation of this study is that detailed intraoperative ventilation parameters, including tidal volume, positive end-expiratory pressure (PEEP), and FiO_2_, were not recorded. Although ventilation strategies were standardized, the absence of these variables precluded a more granular analysis of their potential impact on postoperative pulmonary complications. Future studies incorporating these parameters may further refine risk stratification and improve the predictive value of intraoperative factors. All procedures were performed by a consistent surgical team within a high-volume center and year of surgery, used as a surrogate marker for the learning curve, was not associated with increased pneumonia risk. However, surgeon-specific learning-curve effects were not separately quantified in this study and may still have influenced operative time and perioperative outcomes. Other potential predictive factors, such as sarcopenia, were not included in the analysis [41].

On the other hand, the results are based on a large European cohort reflecting real-world clinical care, supported by the substantial sample size and highly standardized, comparable clinical processes at the center. No patients were excluded due to complications or comorbidities, and outcomes align with international benchmarks. However, differences in patient populations, perioperative management, and surgical expertise may limit generalizability, and external validation in multicenter studies is warranted. In addition, emerging minimally invasive approaches such as Da Vinci Single-Port Robot-Assisted Cervical Esophagectomy (SP RACE) may further refine surgical strategies. By avoiding thoracotomy and thus eliminating the need for lung collapse and one-lung ventilation, this technique may potentially reduce pulmonary stress in selected patients [42,43,44].

In conclusion, prolonged OLV during MIE and RAMIE is associated with an increased risk of postoperative pneumonia, rising by 4% for every 10 min. Pneumonia does not significantly impact overall survival, likely due to effective perioperative management and early diagnosis. OLV duration may serve as a practical intraoperative indicator to guide risk assessment and optimize postoperative care in minimally invasive and robot-assisted Ivor Lewis esophagectomy.

## Figures and Tables

**Table 1:** Demographics, perioperative characteristics, and postoperative parameters.

		Total			MIE			RAMIE			Open/Hybrid			
		619			230			330			59			*p*
Age (years ± SD)		64.3	±	10.6	64.2 ± 10.5			64.6	±	10.7	63.4	±	10.2	*p* = 0.561
Size (cm ± SD)		175	±	0.9	175 ± 0.8			176	±	0.9	175	±	1.0	*p* = 0.800
Weight (kg ± SD)		79.8	±	18.2	79.5 ± 17.8			80.3	±	18.4	78.2	±	19.0	*p* = 0.645
BMI (± SD)		25.8	±	4.9	25.8 ± 4.9			25.9	±	4.9	25.4	±	4.9	*p* = 0.454
Sex (*n*, %)	m	516		83.4%	187		81.3%	281		85.2%	48		81.4%	*p* = 0.442
	f	103		16.6%	43		18.7%	49		14.8%	11		18.6%	
Smoking history (*n*, %)	No	331		53.5%	117		50.9%	180		54.5%	34		57.6%	*p* = 0.279
	Yes	206		33.3%	88		38.3%	102		30.9%	16		27.1%	
	Previous smoking history	82		13.2%	25		10.9%	48		14.5%	9		15.3%	
Diabetes (*n*, %)	No	523		84.5%	192		83.5%	280		84.8%	51		86.4%	*p* = 0.826
	Yes	96		15.5%	38		16.5%	50		15.2%	8		13.6%	
COPD (*n*, %)	Non	571		92.2%	209		90.9%	306		92.7%	56		94.9%	*p* = 0.521
	Yes	48		7.8%	21		9.1%	24		7.3%	3		5.1%	
Previous abdominal surgery (*n*, %)	No	441		71.2%	161		70.0%	247		74.8%	33		55.9%	*p* = 0.011
	Yes	178		28.8%	69		30.0%	83		25.2%	26		44.1%	
Previous thoracic surgery (*n*, %)	No	613		99.0%	227		98.7%	328		99.4%	58		98.3%	*p* = 0.593
	Yes	6		1.0%	3		1.3%	2		0.6%	1		1.7%	
ASA (*n*, %)	1	1		0.2%	0		0.0%	1		0.3%	0		0.0%	*p* = 0.779
	2	268		43.3%	98		42.6%	144		43.6%	26		44.1%	
	3	327		52.8%	120		52.2%	176		53.3%	31		52.5%	
	4	23		3.7%	12		5.2%	9		2.7%	2		3.4%	
Histology (*n*, %)	No viable tumor cells	1		0.2%	1		0.4%	0		0.0%	0		0.0%	*p* = 0.101
	Adeno-Ca	449		72.5%	168		73.0%	243		73.6%	38		64.4%	
	SCC	156		25.2%	57		24.8%	81		24.5%	18		30.5%	
	GIST	1		0.2%	0		0.0%	0		0.0%	1		1.7%	
	Sarcoma	5		0.8%	1		0.4%	2		0.6%	2		3.4%	
	NET	5		0.8%	2		0.9%	3		0.9%	0		0.0%	
	Leiomyoma	1		0.2%	1		0.4%	0		0.0%	0		0.0%	
	Melanoma	1		0.2%	0		0.0%	1		0.3%	0		0.0%	
cUICC-Stage Adeno-Ca (*n*, %)	Stadium I	31		5.1%	14		6.1%	15		4.6%	2		3.4%	*p* = 0.122
	Stadium IIA	6		1.0%	3		1.3%	3		0.9%	0		0.0%	
	Stadium IIB	44		7.2%	17		7.4%	23		7.1%	4		6.8%	
	Stadium III	322		52.7%	122		53.3%	178		55.1%	22		37.3%	
	Stadium IVA	4		0.7%	1		0.4%	3		0.9%	0		0.0%	
	Stadium IVB	26		4.3%	7		3.1%	12		3.7%	7		11.9%	
cUICC-Stage SCC (*n*, %)	Stadium I	11		1.8%	4		1.7%	6		1.8%	1		1.7%	*p* = 0.438
	Stadium II	52		8.4%	20		8.7%	26		7.9%	6		10.2%	
	Stadium III	67		10.8%	23		10.0%	39		11.8%	5		8.5%	
	Stadium IVA	9		1.5%	1		0.4%	5		1.5%	3		5.1%	
	Stadium IVB	4		0.6%	2		0.9%	1		0.3%	1		1.7%	
Neoadjuvant treatment (*n*, %)	No	140		22.6%	61		26.5%	67		20.3%	12		20.3%	*p* = 0.203
	Yes	479		77.4%	169		73.5%	263		79.7%	47		79.7%	
Thoracic OP- duration (min. ± SD)		194.8	±	51.8	186.8	±	55.9	203.5	±	48.3	177.3	±	44.6	*p* < 0.001
Total OP-duration (min. ± SD)		326.0	±	80.2	309.9	±	87.4	334.3	±	74.9	342.0	±	69.5	*p* < 0.001
Abdominal OP- duration (min. ± SD)		132	±	47.94	123	±	50.28	132	±	42.52	165	±	53.28	*p* < 0.001
Procedure (*n*, %)	Ivor Lewis	601		97.1%	222		96.5%	321		97.3%	58		98.3%	*p* = 0.737
	McKeown	18		2.9%	8		3.5%	9		2.7%	1		1.7%	
R-Stadium (*n*, %)	R0	589		95.2%	216		93.9%	317		96.1%	56		94.9%	*p* = 0.506
	R1	30		4.8%	14		6.1%	13		3.9%	3		5.1%	
Hospital stay (d ± SD)		17.2	±	15.6	18.1	±	17.2	16.0	±	13.9	19.9	±	17.2	*p* = 0.002
ICU-stay (d ± SD)		4.4	±	12.7	5.1	±	14.8	3.9	±	11.8	4.4	±	7.4	*p* < 0.001
30d readmission (*n*, %)	No	538		86.9%	196		85.2%	292		88.5%	50		84.7%	*p* = 0.463
	Yes	81		13.1%	34		14.8%	38		11.5%	9		15.3%	
30d mortality (*n*, %)	No	610		98.5%	224		97.4%	328		99.4%	58		98.3%	*p* = 0.148
	Yes	9		1.5%	6		2.6%	2		0.6%	1		1.7%	
Anastomotic leakage (*n*, %)	No	545		88.2%	198		86.5%	297		90.0%	50		84.7%	*p* = 0.306
	Yes	73		11.8%	31		13.5%	33		10.0%	9		15.3%	
Pneumonia (*n*, %)	No	504		81.4%	183		79.6%	270		81.8%	51		86.4%	*p* = 0.463
	Yes	115		18.6%	47		20.4%	60		18.2%	8		13.6%	
Sepsis (*n*, %)	No	597		96.4%	218		94.8%	322		97.6%	57		96.6%	*p* = 0.213
	Yes	22		3.6%	12		5.2%	8		2.4%	2		3.4%	

**Table 2:** Distribution of one-lung ventilation duration for *n* = 619 patients: *n* = 230 MIE, *n* = 330 RAMIE; *a = MIE vs. RAMIE, b = MIE vs. open/hybrid, c = RAMIE vs. open/hybrid. * The surgical approaches show distinct distributions across thoracic operative time intervals.

One-Lung Ventilation [h]	Total	MIE	RAMIE	Open/Hybrid	*p*
	619	230	330	59	
<2 h (*n*, %)	31 (5.0%)	22 (9.6%)	5 (1.5%)	4 (6.8%)	
2–3 h (*n*, %)	229 (37.0%)	87 (37.8%)	107 (32.4%)	35 (59.3%)	*p* < 0.001 *abc
3–4 h (*n*, %)	257 (41.5%)	81 (35.2%)	161 (48.8%)	15 (25.4%)	
>4 h (*n*, %)	102 (16.5%)	40 (17.4%)	57 (17.3%)	5 (8.5%)	

**Table 3:** Incidence of pneumonia according to one-lung ventilation duration.

	Pneumonia		
One-Lung Ventilation [h]	Total (*n*)	Pneumonia Cases (*n*)	Pneumonia Cases (%)
<2 h	31	4	12.9
2–3 h	229	38	16.6
3–4 h	257	47	18.3
>4 h	102	26	25.5
Total	619	115	18.6

## Data Availability

The datasets presented in this article are not readily available due to institutional data protection regulations and the retrospective nature of the study. Requests to access the datasets should be directed to the corresponding author.

## References

[1] Bray F., Laversanne M., Sung H., Ferlay J., Siegel R.L., Soerjomataram I., Jemal A. (2024). Global cancer statistics 2022: GLOBOCAN estimates of incidence and mortality worldwide for 36 cancers in 185 countries. CA Cancer J. Clin..

[2] He Y., Liang D., Du L., Guo T., Liu Y., Sun X., Wang N., Zhang M., Wei K., Shan B. (2020). Clinical characteristics and survival of 5283 esophageal cancer patients: A multicenter study from eighteen hospitals across six regions in China. Cancer Commun..

[3] Morgan E., Soerjomataram I., Rumgay H., Coleman H.G., Thrift A.P., Vignat J., Laversanne M., Ferlay J., Arnold M. (2022). The Global Landscape of Esophageal Squamous Cell Carcinoma and Esophageal Adenocarcinoma Incidence and Mortality in 2020 and Projections to 2040: New Estimates from GLOBOCAN 2020. Gastroenterology.

[4] Sung H., Ferlay J., Siegel R.L., Laversanne M., Soerjomataram I., Jemal A., Bray F. (2021). Global Cancer Statistics 2020: GLOBOCAN Estimates of Incidence and Mortality Worldwide for 36 Cancers in 185 Countries. CA Cancer J. Clin..

[5] Yang H., Wang F., Hallemeier C.L., Lerut T., Fu J. (2024). Oesophageal cancer. Lancet.

[6] Grimminger P.P., Hadzijusufovic E., Babic B., van der Sluis P.C., Lang H. (2020). Innovative fully robotic 4-arm Ivor Lewis esophagectomy for esophageal cancer (RAMIE4). Dis. Esophagus.

[7] Gockel I., Niebisch S., Ahlbrand C.J., Hoffmann C., Möhler M., Düber C., Lang H., Heid F. (2016). Risk and Complication Management in Esophageal Cancer Surgery: A Review of the Literature. Thorac. Cardiovasc. Surg..

[8] Manara M., Bona D., Bonavina L., Aiolfi A. (2024). Impact of pulmonary complications following esophagectomy on long-term survival: Multivariate meta-analysis and restricted mean survival time assessment. Updates Surg..

[9] Chen J., Zhao Y., Yang W., Duan L., Niu L., Li Z., Zhang Y., Miao Y., Fan A., Wei S. (2025). Pulmonary infection after esophageal cancer surgery: Impact on the reality, risk factors and development of a predictive nomogram. World J. Surg. Oncol..

[10] Molena D., Mungo B., Stem M., Lidor A.O. (2014). Incidence and risk factors for respiratory complications in patients undergoing esophagectomy for malignancy: A NSQIP analysis. Semin. Thorac. Cardiovasc. Surg..

[11] Ishikawa S., Ozato S., Ebina T., Yoshioka S., Miichi M., Watanabe M., Yokota M. (2022). Early postoperative pulmonary complications after minimally invasive esophagectomy in the prone position: Incidence and perioperative risk factors from the perspective of anesthetic management. Gen. Thorac. Cardiovasc. Surg..

[12] Lai G., Guo N., Jiang Y., Lai J., Li Y., Lai R. (2020). Duration of one-lung ventilation as a risk factor for postoperative pulmonary complications after McKeown esophagectomy. Tumori.

[13] Low D.E., Alderson D., Cecconello I., Chang A.C., Darling G.E., D’Journo X.B., Griffin S.M., Hölscher A.H., Hofstetter W.L., Jobe B.A. (2015). International Consensus on Standardization of Data Collection for Complications Associated with Esophagectomy: Esophagectomy Complications Consensus Group (ECCG). Ann. Surg..

[14] Rademacher J., Ewig S., Grabein B., Nachtigall I., Abele-Horn M., Deja M., Gaßner M., Gatermann S., Geffers C., Gerlach H. (2025). Epidemiologie, Diagnostik und Therapie erwachsener Patienten mit nosokomialer Pneumonie/Epidemiology, diagnosis and treatment of adult patients with nosocomial pneumonia. Pneumologie.

[15] Schmidt H.M., Gisbertz S.S., Moons J., Rouvelas I., Kauppi J., Brown A., Asti E., Luyer M., Lagarde S.M., Berlth F. (2017). Defining Benchmarks for Transthoracic Esophagectomy: A Multicenter Analysis of Total Minimally Invasive Esophagectomy in Low Risk Patients. Ann. Surg..

[16] Loop T. (2020). One-Lung Ventilation. Anästh. Intensivmed..

[17] Larsen R., Annecke T., Fink T. (2022). Anästhesie.

[18] Dreyfuss D., Saumon G. (1998). Ventilator-induced lung injury: Lessons from experimental studies. Am. J. Respir. Crit. Care Med..

[19] Tekinbas C., Ulusoy H., Yulug E., Erol M.M., Alver A., Yenilmez E., Geze S., Topbas M. (2007). One-lung ventilation: For how long?. J. Thorac. Cardiovasc. Surg..

[20] Sugasawa Y., Yamaguchi K., Kumakura S., Murakami T., Kugimiya T., Suzuki K., Nagaoka I., Inada E. (2011). The effect of one-lung ventilation upon pulmonary inflammatory responses during lung resection. J. Anesth..

[21] Luyer M.D., Greve J.W., Hadfoune M., Jacobs J.A., Dejong C.H., Buurman W.A. (2005). Nutritional stimulation of cholecystokinin receptors inhibits inflammation via the vagus nerve. J. Exp. Med..

[22] Tracey K.J. (2009). Reflex control of immunity. Nat. Rev. Immunol..

[23] Mazzone S.B., Canning B.J. (2013). Autonomic neural control of the airways. Handb. Clin. Neurol..

[24] D’Journo X.B., Michelet P., Marin V., Diesnis I., Blayac D., Doddoli C., Bongrand P., Thomas P.A. (2010). An early inflammatory response to oesophagectomy predicts the occurrence of pulmonary complications. Eur. J. Cardiothorac. Surg..

[25] Weijs T.J., Ruurda J.P., Luyer M.D.P., Cuesta M.A., van Hillegersberg R., Bleys R. (2017). New insights into the surgical anatomy of the esophagus. J. Thorac. Dis..

[26] Chen B., Ke W., Li M. (2025). A nomogram predicting the risk of postoperative pneumonia after esophagectomy in esophageal carcinoma. Front. Med..

[27] Baar W., Semmelmann A., Anselm F., Loop T., Heinrich S., Working Group of the German Thorax Registry (2025). Risk Factors for Postoperative Pulmonary Complications in Patients Undergoing Thoracotomy for Indications Other than Primary Lung Cancer Resection: A Multicenter Retrospective Cohort Study from the German Thorax Registry. J. Clin. Med..

[28] Peters A.K., Juratli M.A., Roy D., Merten J., Fortmann L., Pascher A., Hoelzen J.P. (2023). Factors Influencing Postoperative Complications Following Minimally Invasive Ivor Lewis Esophagectomy: A Retrospective Cohort Study. J. Clin. Med..

[29] Mann C., Jezycki T., Berlth F., Hadzijusufovic E., Uzun E., Mähringer-Kunz A., Lang H., Klöckner R., Grimminger P.P. (2023). Effect of thoracic cage width on surgery time and postoperative outcome in minimally invasive esophagectomy. Surg. Endosc..

[30] Hauge T., Johnson E., Fasting M., Førland D., Skagemo C., Mala T. (2025). From conventional minimally invasive to robotic-assisted Ivor Lewis esophagectomy—A Nordic single-center retrospective study. Eur. J. Surg. Oncol..

[31] Jeon Y.H., Yun J.K., Jeong Y.H., Gong C.S., Lee Y.S., Kim Y.H. (2023). Surgical outcomes of 500 robot-assisted minimally invasive esophagectomies for esophageal carcinoma. J. Thorac. Dis..

[32] Kingma B.F., Hadzijusufovic E., Van der Sluis P.C., Bano E., Lang H., Ruurda J.P., van Hillegersberg R., Grimminger P.P. (2020). A structured training pathway to implement robot-assisted minimally invasive esophagectomy: The learning curve results from a high-volume center. Dis. Esophagus.

[33] Kooij C.D., de Jongh C., Kingma B.F., van Berge Henegouwen M.I., Gisbertz S.S., Chao Y.K., Chiu P.W., Rouanet P., Mourregot A., Immanuel A. (2025). The Current State of Robot-Assisted Minimally Invasive Esophagectomy (RAMIE): Outcomes from the Upper GI International Robotic Association (UGIRA) Esophageal Registry. Ann. Surg. Oncol..

[34] Perry R., Barbosa J.P., Perry I., Barbosa J. (2024). Short-term outcomes of robot-assisted versus conventional minimally invasive esophagectomy for esophageal cancer: A systematic review and meta-analysis of 18,187 patients. J. Robot. Surg..

[35] Banks K.C., Hsu D.S., Velotta J.B. (2022). Outcomes of Minimally Invasive and Robot-Assisted Esophagectomy for Esophageal Cancer. Cancers.

[36] Ekeke C.N., Kuiper G.M., Luketich J.D., Ruppert K.M., Copelli S.J., Baker N., Levy R.M., Awais O., Christie N.A., Dhupar R. (2023). Comparison of robotic-assisted minimally invasive esophagectomy versus minimally invasive esophagectomy: A propensity-matched study from a single high-volume institution. J. Thorac. Cardiovasc. Surg..

[37] Zhang Y., Dong D., Cao Y., Huang M., Li J., Zhang J., Lin J., Sarkaria I.S., Toni L., David R. (2023). Robotic Versus Conventional Minimally Invasive Esophagectomy for Esophageal Cancer: A Meta-analysis. Ann. Surg..

[38] Tanaka K., Yamasaki M., Kobayashi T., Yamashita K., Makino T., Saitoh T., Takahashi T., Kurokawa Y., Nakajima K., Motoori M. (2021). Postoperative pneumonia in the acute phase is an important prognostic factor in patients with esophageal cancer. Surgery.

[39] Nishiyama M., Takeda S., Watanabe Y., Iida M., Yamamoto T., Nakashima C., Matsui H., Shindo Y., Tokumitsu Y., Tomochika S. (2024). Preventing Pneumonia in High-risk Patients After Esophageal Cancer Surgery: Mini-tracheostomy and Tazobactam/Piperacillin. In Vivo.

[40] van de Beld J.J., Crull D., Mikhal J., Geerdink J., Veldhuis A., Poel M., Kouwenhoven E.A. (2024). Complication Prediction after Esophagectomy with Machine Learning. Diagnostics.

[41] Hasegawa K., Wakasa M., Okura K., Takahashi Y., Nagaki Y., Sato Y., Wakita A., Kasukawa Y., Miyakoshi N. (2025). Respiratory Sarcopenia Is Associated with Postoperative Pulmonary Complications in Patients with Esophageal Cancer. J. Surg. Oncol..

[42] Hadzijusufovic E., Lozanovski V.J., Griemert E.-V., Bellaio L., Lang H., Grimminger P.P. (2024). Single-Port da Vinci Robot-Assisted Cervical Esophagectomy: How to Do It. Thorac. Cardiovasc. Surg..

[43] Renger F., Bellaio L., Hadzijusufovic E., Lozanovski V.J., Lang H., Grimminger P.P. (2025). Single-port robot-assisted cervical esophagectomy (SP RACE): Combining precision mediastinal lymphadenectomy and complete extrapulmonary dissection. JTCVS Tech..

[44] Lozanovski V.J., Bellaio L., Hadzijusufovic E., Meier O., Renger F., Wandhoefer C., Gisbertz S.S., van Hillegersberg R., Lang H., Peter P. (2026). First series of da Vinci single-port robotic-assisted cervical oesophagectomy: Single-centre IDEAL stage 2a/2b study. BJS Open.

